# Treatment with Gac Fruit Extract and Probiotics Reduces Serum Trimethylamine N-Oxide in Chronic Kidney Disease Rats

**DOI:** 10.3390/nu16172997

**Published:** 2024-09-05

**Authors:** Panumas Kamkang, Pakkapon Rattanachaisit, Weerapat Anegkamol, Mana Taweevisit, Suwimol Sapwarobol, Somying Tumwasorn, Natthaya Chuaypen, Thasinas Dissayabutra

**Affiliations:** 1 Metabolic Disease in Gastrointestinal and Urinary System Research Unit, Department of Biochemistry, Faculty of Medicine, Chulalongkorn University, Bangkok 10330, Thailand; panumas.k@chula.ac.th (P.K.); weerapat.a@chula.ac.th (W.A.); natthaya.c@chula.ac.th (N.C.); 2 Department of Physiology, Faculty of Medicine, Chulalongkorn University, Bangkok 10330, Thailand; pakkapon.r@chula.ac.th; 3 Department of Pathology, Faculty of Medicine, Chulalongkorn University, Bangkok 10330, Thailand; mana.t@chula.ac.th; 4 The Medical Food Research Group, Department of Nutrition and Dietetics, Faculty of Allied Health Sciences, Chulalongkorn University, Bangkok 10330, Thailand; suwimol.sa@chula.ac.th; 5 Department of Microbiology, Faculty of Medicine, Chulalongkorn University, Bangkok 10330, Thailand; somying.t@chula.ac.th

**Keywords:** chronic kidney disease, gac fruit, probiotics, TMAO, gut microbiota

## Abstract

Chronic kidney disease (CKD) affects more than 850 million people worldwide, contributing to morbidity and mortality, particularly through cardiovascular disease (CVD). The altered composition in CKD patients leads to increased production and absorption of uremic toxins such as trimethylamine (TMA) and its oxidized form, trimethylamine N-oxide (TMAO), which are associated with cardiovascular risks. This study investigated the potential of supplementary interventions with high-carotenoid-content gac fruit extract and probiotics to mitigate serum TMAO by modulating the gut microbiota. We conducted an animal study involving 48 male Wistar rats, divided into six groups: the control, CKD control, and four treatment groups receiving gac fruit extract, carotenoid extract, or combinations with *Ligilactobacillus salivarius* and *Lactobacillus crispatus* and *Lactobacillus casei* as a standard probiotic. CKD was induced in rats using cisplatin and they were supplemented with choline to enhance TMA production. The measures included serum creatinine, TMAO levels, gut microbiota composition, and the expression of fecal TMA lyase and intestinal zonula occluden-1 (ZO-1). CKD rats showed increased TMA production and elevated serum levels of TMAO. Treatment with gac fruit extract and selective probiotics significantly altered the composition of the gut microbiota by decreasing *Actinobacteriota* abundance and increasing the abundance of *Bacteroides*. This combination effectively promoted *ZO-1* expression, reduced fecal TMA lyase, and subsequently lowered serum TMAO levels, demonstrating the therapeutic potential of these interventions. Our results highlight the benefits of gac fruit extract combined with probiotics for the effective reduction in serum TMAO levels in rats with CKD, supporting the further exploration of dietary and microbial interventions to improve outcomes in patients with CKD.

## 1. Introduction

Chronic kidney disease (CKD) is a global health problem affecting more than 850 million people around the world, leading to significant morbidity and mortality [1]. In CKD, alterations in the composition and function of the gut microbiota can lead to increased production and absorption of uremic toxins. This gut dysbiosis, a disturbance of the gastrointestinal microbial balance, has been linked to the progression of CKD and its complications, especially cardiovascular diseases (CVD). 

Among the myriad factors that contribute to the progression and exacerbation of CKD and CVD, the gut microbiota and its metabolic by-products, notably trimethylamine (TMA) and its oxidized form, trimethylamine N-oxide (TMAO), have emerged as critical players. Dietary choline, phosphatidylcholine, and L-carnitine are metabolized by specific gut microbiota to produce TMA, which is then absorbed into the bloodstream and transported to the liver [2]. In the liver, TMA is oxidized by flavin-containing monooxygenase 3 enzyme (FMO3) to form TMAO. Emerging evidence has highlighted TMAO as a proatherogenic molecule, with elevated plasma levels associated with the progression of CKD [3]. Elevated TMAO levels have been associated with atherosclerosis, inflammation, hypertension, and endothelial dysfunction, underscoring their role in the increased cardiovascular morbidity and mortality observed in patients with CKD [4,5].

Probiotics, defined as live microorganisms that confer a health benefit on the host when administered in adequate amounts, have emerged as a promising therapeutic option to restore intestinal microbial balance and mitigate the adverse effects associated with dysbiosis. By modulating the gut microbiota, probiotics have the potential to reduce the production and systemic absorption of uremic toxins, decrease inflammation, and improve intestinal barrier function, thus offering a multifaceted approach to the treatment of CKD [6,7].

Furthermore, carotenoids such as lycopene and beta-carotene have been demonstrated to effectively regulate gut dysbiosis. These compounds encourage the proliferation of beneficial bacteria and suppress the growth of harmful ones, modulate the immune response, diminish oxidative stress, and reduce inflammation in the intestinal epithelium, which can lead to leaky gut syndrome. Carotenoid supplementation can also aid in lowering serum levels of TMAO. The gac fruit (*Momordica cochinchinensis*), a regional plant from Southeast Asia with a high carotenoid content [8], was used in this study as a carotenoid source to enhance the effects of the chosen probiotics in reducing serum TMAO levels in chronic kidney disease (CKD) scenarios.

This study aimed to explore the burgeoning field of the use of probiotics and gac fruit extracts in CKD management, focusing on the reduction in TMAO levels as a novel therapeutic target to mitigate cardiovascular risks and slow renal function decline. By delving into the mechanisms through which probiotics may influence TMAO metabolism and examining the current evidence base for their efficacy in CKD, we aim to illuminate the potential of probiotics as an adjunctive treatment in the comprehensive management of patients with CKD. 

## 2. Materials and Methods

### 2.1. Animal Study

Forty-eight 6-week-old male Wistar rats, purchased from Siam Nomura International Co., Ltd. (Bangkok, Thailand), underwent a one-week acclimatization period before being housed in the Laboratory Animal Unit at the Faculty of Medicine, Chulalongkorn University. The sample size determination was calculated by GPower version 3.1 program. The laboratory environment was meticulously controlled, maintaining a temperature of 25 °C, a relative humidity between 30 and 50%, and a consistent 12 h light/dark cycle. Throughout the study, rats had unrestricted access to food and water. The rats were housed in groups of four per cage in standard polycarbonate cages, each equipped with a stainless-steel wire lid, and a bedded floor lined with wood shavings to provide a comfortable and hygienic environment. The cages were regularly cleaned and sanitized to maintain optimal hygiene conditions. Subsequently, rats were randomly divided into six groups, with eight rats in each group, including the naive control group (control), the CKD-induced rat control group (CKD), CKD-induced rats fed with 6 mg of gac fruit extract (GacCar), CKD rats fed with 6 mg of carotenoid extract (lycopene and beta-carotene) (STDCar), CKD rats fed with 10^9^ CFU of *Ligilactobacillus salivarius* and *Lactobacillus crispatus* and 6 mg of gac fruit extract (GacPro), and CKD rats fed with 10^9^ CFU of *Lactobacillus casei* as a standard probiotic with 6 mg of carotenoid extract (STDPro). 

All CKD rats were injected with cisplatin at a dose of 4 mg per kg body weight 2 weeks before the initiation of the experiment to induce kidney injury. Subsequently, 1% choline (Healthy Hangzhou Husbandry Sci-tec, Hangzhou, China) and 0.5% phosphoric acid were added to the drinking water as an additional source of dietary phosphate and adenine to all groups, including control rats. Probiotic cocktails were supplemented from the 6th to the 15th week of the experiment. In this study, no specific humane endpoints were established. All procedures were conducted in accordance with institutional guidelines for the care and use of laboratory animals to minimize discomfort and stress. Throughout the study, rats were monitored daily for general health and well-being, including signs of distress, pain, abnormal behavior, changes in body weight, and overall physical condition. Isoflurane was implemented to minimize pain and discomfort for the rats during sample collection. Any animal exhibiting signs of severe distress or illness would have been euthanized according to the guidelines set by the institutional animal care and use committee (IACUC). However, no animals in this study required early euthanasia. At the end of the experimental period, all rats were fasted overnight but allowed access to water. Euthanasia was induced via overdosed isoflurane inhalation. Approximately 5 mL of blood was collected from each rat. Blood samples were immediately transferred to heparinized tubes. The tubes were then centrifuged at 1500 RCF for 10 min at 25 °C to separate the plasma and were stored at −80 °C for further biochemical analyses. Feces samples were collected directly from the rectum and were stored in DNA/RNA Shield^TM^ solution (Zymo Research Corporation, Tustin, CA, USA) to preserve nucleic acids and maintain sample integrity for gut microbiota analysis and TMA lyase measurement via qRT-PCR. The heart, aorta, and jejunum of the rats were collected and immediately preserved in 4% paraformaldehyde (PFA) for subsequent analysis. 

### 2.2. Measurement of Biochemical Indicators

Serum creatinine was measured using the automated Alinity ci system at the Department of Laboratory Medicine, Faculty of Medicine, Chulalongkorn University. 

Serum TMAO was measured using the spectrophotometric colorimetric method [9]. Briefly, 100 µL of plasma was added to each well of a 96-well plate along with 20 µL of HCl and 20 µL of manganese dioxide nanoparticles coated with polyelectrolyte polyallylamine hydrochloride (PAH@MnO_2_ NPs). Then, 20 µL of the above mixture was extracted and mixed with 100 µL of the TMB/H_2_O_2_ solution. The resulting spectrophotometry values at 650 nm absorbance (A650) values were recorded to determine the concentration of TMAO in the blood of normal or CKD rats, using comparative adjustment graphs obtained from the testing.

### 2.3. Measurement of TMA Lyase (CutC)

The expression of TMA lyase from fecal extraction was assessed using the QuantStudioTM 5 Real-Time PCR System through quantitative polymerase chain reaction (qPCR) analysis [10]. The primer sequences for TMA lyase and 16S rRNA are detailed in Table 1. For each reaction, a 15 µL volume mixture was prepared, comprising 3.75 µL of SYBR Green, 0.6 µL of the forward primer, 0.6 µL of the reverse primer, 9.95 µL of water for PCR, and 1 µL of cDNA.

The temperatures and durations for the various stages of the qPCR reaction included the hold stage, where the temperature was maintained at 95 °C for 10 min. Subsequently, in the PCR stage, cycles occurred at 95 °C for 30 s, 60 °C for 30 s, and 72 °C for 20 s. The melt curve stage involved steps at 95 °C for 1 s, 60 °C for 20 s, and again 95 °C for 1 s. It is important to note that the TMA lyase underwent 45 cycles at 54 °C.

### 2.4. Determination of Vascular Calcification

In summary, the process involved deparaffinizing samples from the abdominal aorta and aortic arch by immersing them in xylene for 3 min each. This was followed by rehydration in 95% alcohol with multiple dips and then in tap water for 1 min.

For histological analysis, tissues were fixed in 4% formaldehyde, embedded in paraffin, and cut into 5 µm slices. The slices were stained with Von Kossa, which involved using 5% silver nitrate and nuclear fast red. Subsequently, the samples were rinsed with distilled water for 3 min and exposed to bright sunlight or a UV lamp for 1 h. After another 3 min rinse with distilled water, a 5% sodium thiosulfate solution was applied for 1 min, followed by a final 3 min rinse with distilled water. The samples were then counterstained with nuclear fast red for 10 min and given a final rinse with distilled water for 3 min. Finally, the samples were incubated at 60 °C for 5 min.

### 2.5. Immunocytochemistry to Determine Intestinal Zonula Occludens-1 

Immunohistochemical staining with zonula occludens-1 (ZO-1) antibodies was performed using an automated system and the UltraView Universal DAB Detection kit [11]. The anti-ZO-1 antibody utilized was a rabbit monoclonal recombinant antibody (Catalog No. 33-9100, Thermo Fisher Scientific, Waltham, MA, USA). The slides were deparaffinized and rehydrated at 72 °C, followed by deproteinization with the protease 2 enzyme. The slides were treated with inhibitors to block nonspecific binding, followed by incubation with primary ZO-1 antibodies. After washing, secondary horseradish peroxidase (HRP) multimer antibodies were added, followed by signal amplification with diaminobenzidine (DAB) and peroxides. Copper was added to intensify the color, and unstained areas were counterstained with hematoxylin. Three sections of intestinal tissue per rat were analyzed and scored.

To score the ZO-1 expression, we categorized it into five grades, as follows: 0—expression of ZO-1 less than 10%; 1—10–25% expression; 2—26–50% expression; 3—51–75% expression; and 4—more than 75% expression. The ZO-1 expression scores were reported as the median with the interquartile range (IQR).

### 2.6. Statistical Analysis

The data analysis of the experimental animals was performed using GraphPad Prism Version 9.4.1 and SPSS version 23 (IBM statistic, Armonk, NY, USA). The analysis included evaluating weight, creatinine levels, heart weight-to-body weight ratio, TMAO levels, lycopene level, beta-carotene level, and expression of TMA lyase enzyme. Student’s *t*-test was used for comparing between two groups and multiple repeated measures ANOVA was used for comparing various parameters between more than two experimental groups, with a confidence level of 95% (*p* value < 0.05).

Analysis of gut microbiota diversity was performed using the QIIME2 software package version 2024.2. This analysis involved assessing alpha-diversity, beta-diversity, and relative abundance using nonparametric statistical methods. Differences in microbial composition were examined through sample distribution analysis using principal coordinate analysis (PCoA), and statistical analysis was performed using permutational multivariate analysis of variance (PERMANOVA). Additionally, SPSS version 23 (IBM statistic, USA) was used for further analysis.

### 2.7. Ethical Considerations

This research was approved by the Animal Care and Use Committee (CU-ACUC) (Protocol No. 005/2565, approval date: 1 September 2022) and the MDCU Institutional Biosafety Committee (Protocol No. 006/2022, approval date: 26 July 2022), All experiments were carried out in accordance with the IACUC and ARRIVE guidelines and regulations.

## 3. Results

### 3.1. Body Weight and Heart Weight

**Figure 1:**
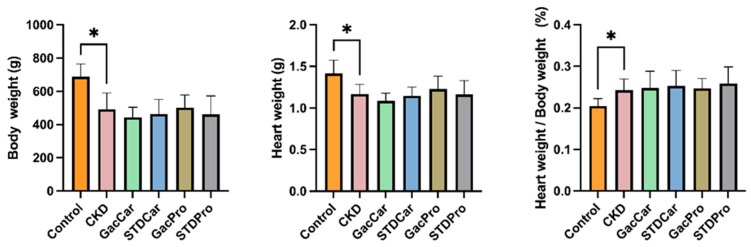
Body weight, heart weight and heart weight per body weight of experimental rats. The weight was measured at the end of the study. Values are presented as mean ± standard deviation (*n* = 8). * *p* < 0.05 vs. control. CKD: chronic kidney disease; GacCar: gac fruit extract supplemented; STDCar: standard carotenoid extract supplemented; GacPro: gac fruit extract with *Ligilactobacillus salivarius* and *Lactobacillus crispatus* supplemented; STDPro: *lactobacillus casei* supplemented.

All CKD-induced rats had a lower body weight than the control at the end of the experiment (Figure 1). Similarly, the heart weights of all CKD rats were lower than those of the control (Figure 1). However, the heart weight-to-body weight ratio of CKD rats was significantly higher than that of their non-CKD counterparts (Figure 1). There were no differences in body weight, heart weight, and heart weight-to-body weight ratio between the groups of CKD rats.

### 3.2. Uremic Toxin

**Figure 2:**
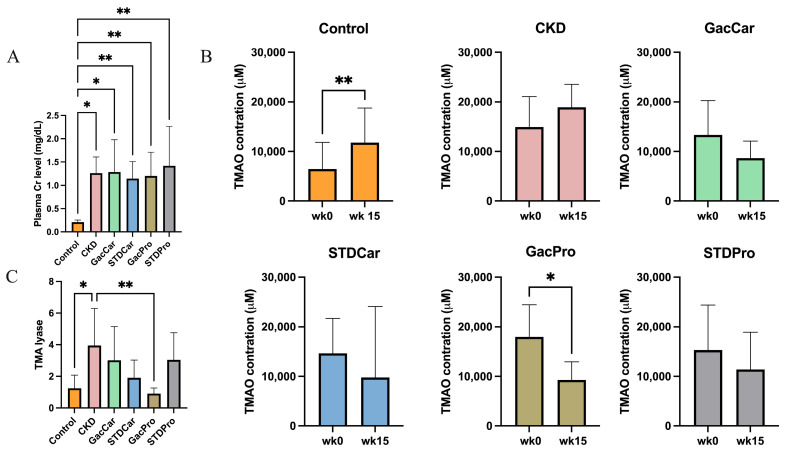
Plasma uremic toxins and TMA lyase expression. (**A**) Plasma creatinine at week 15. (**B**) Comparison between serum TMAO at week 0 and week 15 in each experimental group. (**C**) Fecal TMA lyase RNA expression in week 15. Values are presented as mean ± standard deviation (*n* = 8). * *p* < 0.05; ** *p* < 0.01.

Plasma creatinine levels were highly elevated in CKD rats compared to the control group (Figure 2A). Serum TMAO levels at the start of the experiment were significantly lower in the control group compared with the CKD rats, but subsequently increased at the end of the study, presumably due to the ingestion of excessive choline (Figure 2B). Among the CKD groups, only the GacPro group showed a significant decrease in serum TMAO level at week 15. 

Furthermore, overexpression of fecal TMA lyase was observed in the CKD and STDPro groups compared to the control group (Figure 2C). However, among CKD rats, only GacPro rats had significantly lower expression of TMA lyase compared to the CKD group.

### 3.3. Histopathology of Cardiovascular Tissues and Immunohistochemistry of the Intestinal Tract

The pathological study of the left ventricular cardiac muscle showed no differences in the size of the cardiomyocytes between each group, and no fibrosis was detected (Figure S1). No atherosclerotic plaque nor vascular calcification was observed in the aortic arch (Figure S2) or the abdominal aorta (Figure S3) in any of the groups.

**Figure 3:**
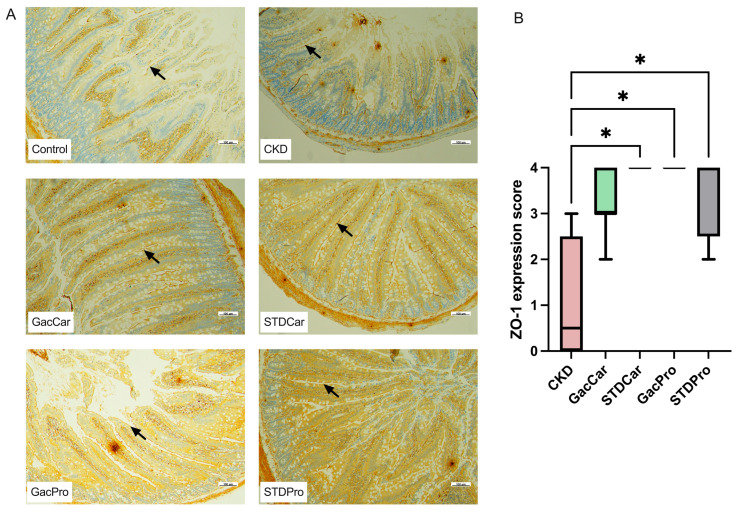
Intestinal zonula occludens type 1 (ZO-1) expression. (**A**) The immunohistochemistry of ZO-1 in the colon. (**B**) Comparison of the ZO-1 expression score between each group. 0: Expression of ZO-1 less than 10%; 1: 10–25% expression; 2: 26–50% expression; 3: 51–75% expression and 4: more than 75% expression. The ZO-1 expression scores are presented as the median with the interquartile range (*n* = 8). * *p* < 0.05 vs. CKD group. Arrow: immunohistochemical staining of the ZO-1 protein.

Immunohistochemistry of the colon demonstrated decreased expression of ZO-1 protein in the CKD groups compared to the control group (Figure 3A). CKD rats treated with standard carotenoids (STDCar) or gac fruit extract combined with probiotics (GacPro) displayed normalized ZO-1 expression, while isolated gac fruit extract (GacCar) or standard probiotics with carotenoid extract (STDPro) could partially rescue the expression of ZO-1 (Figure 3B).

### 3.4. Gut Microbiome Studies

**Figure 4:**
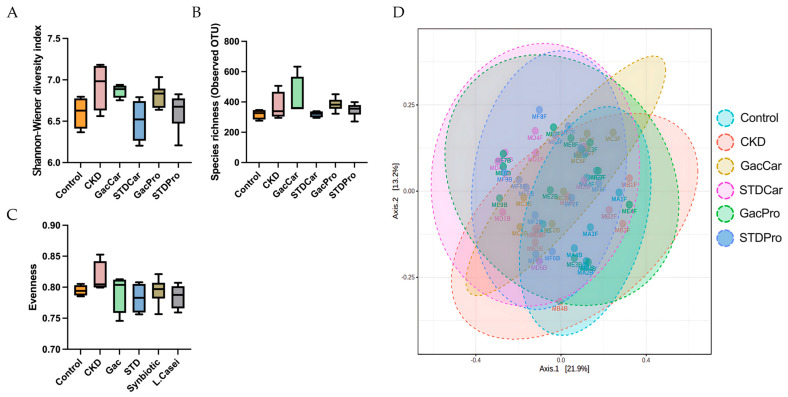
Fecal gut microbiome studies. The gut microbiome was determined using the 16sRNA sequencing method at the end of the study. (**A**) Shannon’s diversity index. (**B**) Richness. (**C**) Pielou’s evenness. (**D**) Bray–Curtis principal coordinate analysis (PCoA) of the fecal microbiota. Box and whisker plot showing the distribution of Shannon’s diversity index, richness, and Pielou’s evenness for each group. The boxes represent the interquartile range (IQR), with whiskers extending to the smallest and largest values.

At the end of the study, although the GacCar and GacPro groups tended to show a limited increase in gut microbiota diversity, no differences in the Shannon index, richness, or Pielou evenness were detected among the groups (Figure 4A–C). In a comparison of diversity, richness, and evenness between week 0 and week 15, only the GacPro rats exhibited an increase in fecal gut microbiota richness at week 15 (Figure S4). However, the Bray–Curtis PCoA results showed significant differences between each group, suggesting that the treatment affected the relative abundance of gut microbiota taxa.

Further analysis of gut microbiota alterations between week 15 and week 0 revealed a remarkable reduction in the decreased relative abundance of *Actinobacteriota* in CKD rats compared to the control (Table 2). An increased relative abundance of *Muribaculaceae*, *Colidextribacter*, and UCG008 and a decreased relative abundance of *Bacteroides* and *Phascolarctobacterium* were observed in CKD rats (Table 3).

The isolated gac fruit extract supplement (GacCar) relatively increased the relative abundance of the phyla *Desulfobacterota* and *Elusimicrobiota* and the genera *Bacteroides*, *Phascolarctobacterium*, and *Bacteroides* in the microbiota. Meanwhile, carotenoid treatment (STDCar) relatively enriched the phyla *Desulfobacterota*, *Elusimicrobiota*, and *Verrucomicrobiota* and the genera *Akkermansia* and *Prevotellaceae_NK3B31_group*.

Gac fruit extract with probiotic treatment (GacPro) reduced the relative abundance of *Actinobacteriota* to a similar level as the control group. In addition, GacPro increased the relative abundance of the phyla *Desulfobacterota* and *Campylobacterota*, and the genera *Bacteroides* and *Prevotellaceae_NK3B31_group*. Therefore, *L. casei* supplementation (STDPro) could only increase the abundance of the *Campylobacterota* phylum and the *Bacteroides* genus.

## 4. Discussion

Atherosclerosis and vascular calcification, a condition characterized by the pathological deposition of calcium and phosphate crystals within the vascular walls, are of critical concern in the management of chronic kidney disease (CKD) patients due to their association with increased cardiovascular mortality. The 2017 KDIGO Clinical Practice Guideline Update for the Diagnosis, Evaluation, Prevention, and Treatment of CKD-MBD highlighted the importance of reducing serum phosphate levels while avoiding the induction of hypercalcemia to mitigate atherosclerosis and vascular calcification risks [12]. This guideline recommends against the use of traditional calcium-based phosphate binders and vitamin D, favoring newer treatments like non-calcium phosphate binders and calcimimetics. Additionally, emerging therapies such as vitamin K and intravenous phytate have been proposed for vascular calcification prevention, though their efficacy and safety require further investigation [13,14]. In response to these challenges, we developed a probiotic approach aimed at reducing serum TMAO levels to slow the progression of cardiac dysfunction, atherosclerosis and vascular calcification.

TMAO, a uremic toxin, is currently considered one of the main culprits in cardiovascular disease and renal progression in CKD patients [15]. Several mechanisms have been described in the elevation of serum TMAO, including increased intestinal TMA production, absorption, liver conversion to TMAO, and decreased elimination of TMAO.

The production of TMA in the gut is primarily facilitated by certain bacteria that possess the metabolic ability to convert dietary precursors such as choline, phosphatidylcholine, and L-carnitine into TMA. *Firmicutes*, particularly *Peptostreptococcaceae* and *Clostridiaceae*, together with *Proteobacteria* and *Actinobacteria*, are efficient in converting choline and L-carnitine to TMA [16] due to their ability to produce TMA-converting enzymes, particularly TMA lyase (*CutC*), a crucial enzyme in TMA synthesis [17,18]. Patients with CKD typically have an increased abundance of Proteobacteria and Actinobacteria [19]. The *Firmicutes/Bacteroides* (F/B) ratio was altered in CKD patients, but this could have been influenced by other confounders. Chen, R. (2022) revealed that Firmicutes were the most prevalent in diabetic CKD patients, while Bacteroides were more abundant in non-diabetic CKD patients [20].

Gut dysbiosis in CKD, including increased *Proteobacteria* and *Actinobacteria* abundance and an imbalance in the F/B ratio, is associated with macrophage activation, exacerbating a chronic low-grade inflammatory state and a low level of short-chain fatty acid (SCFA) production and hypertension [21]. This dysbiosis damages intestinal epithelial cells and disrupts the intestinal barrier, causing gut leakage, which improves TMA absorption. In the liver, TMA is converted to TMAO by flavin-containing monooxygenase 3 (FMO3). In CKD, FMO3 activity is enhanced and TMAO production is augmented [22]. Additionally, TMAO is excreted in the urine; thus, CKD patients with a lower glomerular filtration rate have a higher serum TMAO due to inefficient elimination [23]. 

To alleviate renal progression and cardiovascular complications, a multifaceted approach was studied for its potential to modulate TMAO. Iodomethylcholine, a non-lethal inhibitor of gut microbial trimethylamine (TMA) production, could reduce renal injury indices and vascular inflammation markers in adenine-induced CKD rats [24,25]. Other agents, such as GLP-1 receptor agonists and TMA lyase inhibitors, can modulate the gut microbiota and circulating TMAO levels [18,24]. In this study, our aim was to use high-carotenoid-content gac fruit extract combined with probiotics to suppress circulating TMAO levels. 

CKD was induced in rats using cisplatin, and with additional choline ingestion they developed gut dysbiosis, including increased *Actinobacteriota* abundance, decreased *Bacteroides* and *Phascolarctobacterium* abundance, decreased intestinal ZO-1 expression, and increased fecal TMA lyase expression. Serum creatinine and TMAO levels in CKD rats were high, but no myocardial fibrosis or vascular calcification was observed in the great vessels. Supplementation of gac fruit extract with *Ligilactobacillus salivarius* and *Lactobacillus crispatus* effectively rescued gut dysbiosis and ZO-1 expression. We observed that lowering the abundance of *Actinobacteriota*, one of the main TMA lyase-containing commensal gut microbiota, efficiently restricted intestinal TMA production, as we found the down-regulation of fecal TMA lyase and reduced serum TMAO levels in GacPro rats with CKD. Although supplementation with carotenoids, gac fruit extract with a high carotenoid content, or a *Lactobacillus casei* probiotic partially corrected gut dysbiosis, increasing the abundance of *Bacteroides*, the abundance of *Actinobacteriota* did not increase, and there was no effect on serum TMAO. 

In the present study, TMAO levels in all CKD rat groups at the initiation of the study were higher than those in the control rats; however, only the levels in rats treated with gac fruit extract combined with probiotics significantly reduced. We assumed that probiotic treatment plays a significant role in the modulation of the gut microbiota and promotion of the gut barrier. Some researchers are convinced that TMAO-regulating probiotics are strain-specific [6]. *L. rhamnosus GG* showed the ability to affect serum TMAO in both animals and humans [26], while supplementation with *L. plantarum* ZDY04, *E. aerogenes* ZDY01, *L. plantarum* LP1145, *L. amylovorus,* and *B. Longum Subsp. Longum BL21* reduced serum TMAO in animals, but it was not effective in CKD patients [27,28]. Our results added *L. salivarius* and *L. crispatus* to the arsenal.

Carotenoids promote intestinal health by increasing commensal bacterial proliferation and strengthening the intestinal barrier. Carotenoids at low levels have antioxidant properties and regulate the intestinal immunity of the host [29]. Most of the carotenoids ingested remain in the digestive tract and are metabolized by the gut microbiota [30]. Carotenoids can modulate the gut microbiota, such as the F/B ratio in obesity, as well as *Proteobacteria*, *Faecalibacteria*, and *Prevotella* in models of alcoholic fatty liver disease and colitis [31,32], and particularly promote the growth of *Bifidobacteria* and *Lactobacilli* [33]. Some research has revealed that a high dietary intake of carotenoids is associated with a lower CVD mortality rate in CKD patients [34,35,36], mainly due to their antioxidant and anti-inflammatory effects on the kidneys [37]. 

It was noted that a significantly increased level of TMAO was obtained in control rats without CKD development, as indicated by the normal serum creatinine levels at the end of the study. We assumed that this could be the result of excess choline ingestion, because control rats continued to have a normal appetite and had higher amounts of food intake per day than all CKD rats. A previous study showed that non-CKD rats with high choline intake developed high serum TMAO levels, tubulointerstitial fibrosis, and collagen deposition [38].

Our results demonstrate that the combination of selected *Ligilactobacillus salivarius* and *Lactobacillus crispatus* with gac fruit extract, which contains high levels of carotenoids, had synergistic effects and can be used as an adjuvant agent in a supplement to reduce serum TMAO. This is significant because TMAO is a uremic toxin that causes cardiovascular and renal complications in patients with CKD.

## 5. Conclusions

We demonstrated that interventions such as the use of gac fruit extract rich in carotenoids, in combination with specific probiotics such as *Ligilactobacillus salivarius* and *Lactobacillus crispatus*, can reduce the serum levels of TMAO by mitigating gut dysbiosis, promoting the gut barrier, and suppressing the expression of the TMA lyase enzyme. Hence, probiotic supplementation with gac fruit extract potentially lowers the risk of CKD progression and associated cardiovascular complications. 

## Figures and Tables

**Table 1:** Quantitative real-time PCR primer sequences.

Target	Primer	Sequence	Product Size (bp)
TMA lyase	Forward	TTYGCIGGITAYCARCCNTT	275
	Reverse	TGNGGYTCIACRCAICCCAT	
16S rRNA	Forward	AGRGTTHGATYMTGGCTCAG	177
	Reveres	TGCTGCCTCCCGTAGGAGT	

**Table 2:** Relative abundance decreases in the gut microbiota (week 15 versus week 0) between CKD rats and other groups.

Comparison Group	Phylum/Genus Level	Bacteria	Change in Relative Abundance (Week 15 vs. Week 0)		*p* Value
			CKD	Comparison Group	
CKD vs. Control	Phylum	*Actinobacteriota*	−0.062 (0.53)	−0.226 (0.38)	0.043 *
	Genus	*Muribaculaceae*	2.355 (7.24)	−2.310 (6.05)	0.021 *
	Genus	*Colidextribacter*	0.829 (2.90)	0.716 (0.81)	0.043 *
	Genus	UCG008	0.379 (0.80)	−0.071 (0.49)	0.021 *
CKD vs. GacPro	Phylum	*Actinobacteriota*	−0.062 (0.53)	−0.377 (0.43)	0.011 *

Data showed in mean ± interquartile range, * *p* < 0.05 compare with CKD group.

**Table 3:** Relative abundance increases (week-15 versus week-0) between CKD rats and other groups.

Comparison Group	Phylum/Genus Level	Bacteria	Change in Relative Abundance (Week 15 vs. Week 0)		*p* Value
			CKD	Comparison Group	
CKD vs. Control	Genus	*Bacteroides*	−1.190 (3.66)	0.851 (1.39)	0.021 *
	Genus	*Phascolarctobacterium*	−0.346 (2.25)	1.098 (2.08)	0.021 *
CKD vs. GacCar	Phylum	*Desulfobacterota*	−0.367 (0.47)	0.204 (0.59)	0.021 *
	Phylum	*Elusimicrobiota*	0.020 (1.21)	2.464 (3.31)	0.043 *
	Genus	*Bacteroides*	−1.190 (3.66)	−0.376 (0.19)	0.021 *
	Genus	*Phascolarctobacterium*	−0.346 (2.25)	1.098 (2.08)	0.021 *
	Genus	*Bacteroides*	−1.190 (3.66)	−0.191 (0.59)	0.021 *
CKD vs. STDCar	Phylum	*Verrucomicrobiota*	−0.7660(1.11)	0.296 (1.05)	0.021 *
	Phylum	*Desulfobacterota*	−0.367 (0.47)	0.336 (1.50)	0.021 *
	Phylum	*Elusimicrobiota*	0.020 (1.21)	0.027 (1.56)	0.021 *
	Genus	*Prevotellaceae_NK3B31_group*	−6.089 (2.67)	−2.949 (4.05)	0.021 *
	Genus	*Akkermansia*	−0.780 (1.11)	0.296 (1.05)	0.021 *
CKD vs. GacPro	Phylum	*Desulfobacterota*	−0.367 (0.47)	0.275 (0.36)	0.011 *
	Phylum	*Campylobacterota*	0.014 (0.14)	0.283 (0.44)	0.033 *
	Genus	*Bacteroides*	−1.190 (3.66)	−0.027 (1.41)	0.011 *
	Genus	*Prevotellaceae_NK3B31_group*	−6.089 (2.67)	0.221 (3.19)	0.019 *
CKD vs. STDPro	Phylum	*Campylobacterota*	0.014 (0.14)	0.357 (0.24)	0.011 *
	Genus	*Bacteroides*	−1.190 (3.66)	−0.090 (1.28)	0.011 *

Data shown as mean ± interquartile range. * *p* < 0.05 compared with the CKD group.

## Data Availability

The data presented in this study are available upon request.

## Supplementary Materials

The following supporting information can be downloaded at: https://www.mdpi.com/article/10.3390/nu16172997/s1, Figure S1: Pathology of left ventricular cardiomyocytes (H&E staining, 40× light microscopy); Figure S2: Pathology of aortic arch (von Kossa staining, 40× light microscopy); Figure S3: Pathology of abdominal aorta (von Kossa staining, 40× light microscopy); Figure S4: The comparison of fecal gut microbiota richness between week 0 vs. week 15.
