# Supplementary material for: Treatment with Gac Fruit Extract and Probiotics Reduces Serum Trimethylamine N-Oxide in Chronic Kidney Disease Rats
Source: Nutrients. 2024 Sep 5;16(17):2997. doi: 10.3390/nu16172997 (PMC11396870; doi:10.3390/nu16172997)
Supplement: Supplementary file 1 [file nutrients-16-02997-s001.zip › nutrients-3171936-supplementary.pdf]

Supplementary

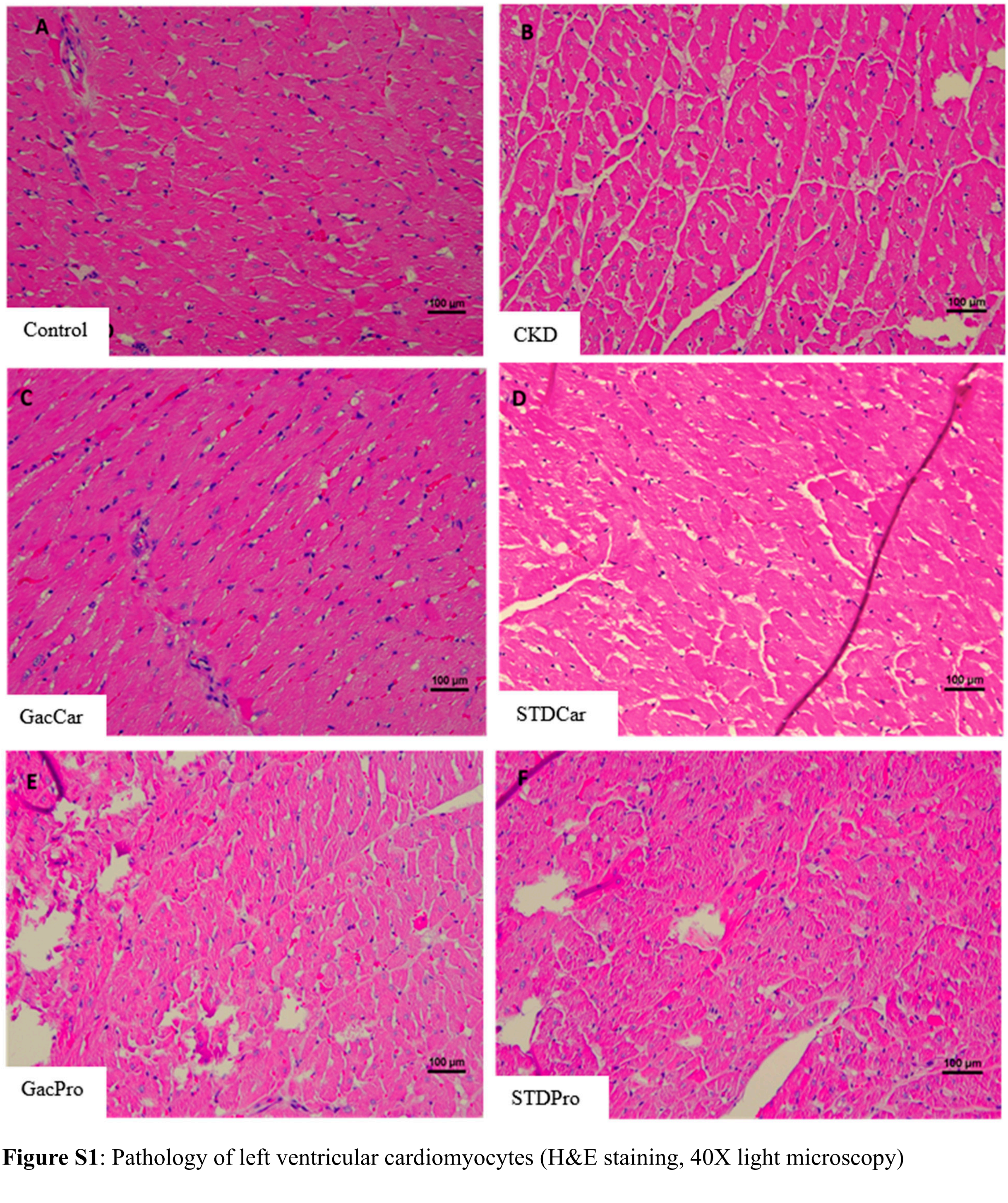

Figure S1: Pathology of left ventricular cardiomyocytes (H&E staining, 40X light microscopy)

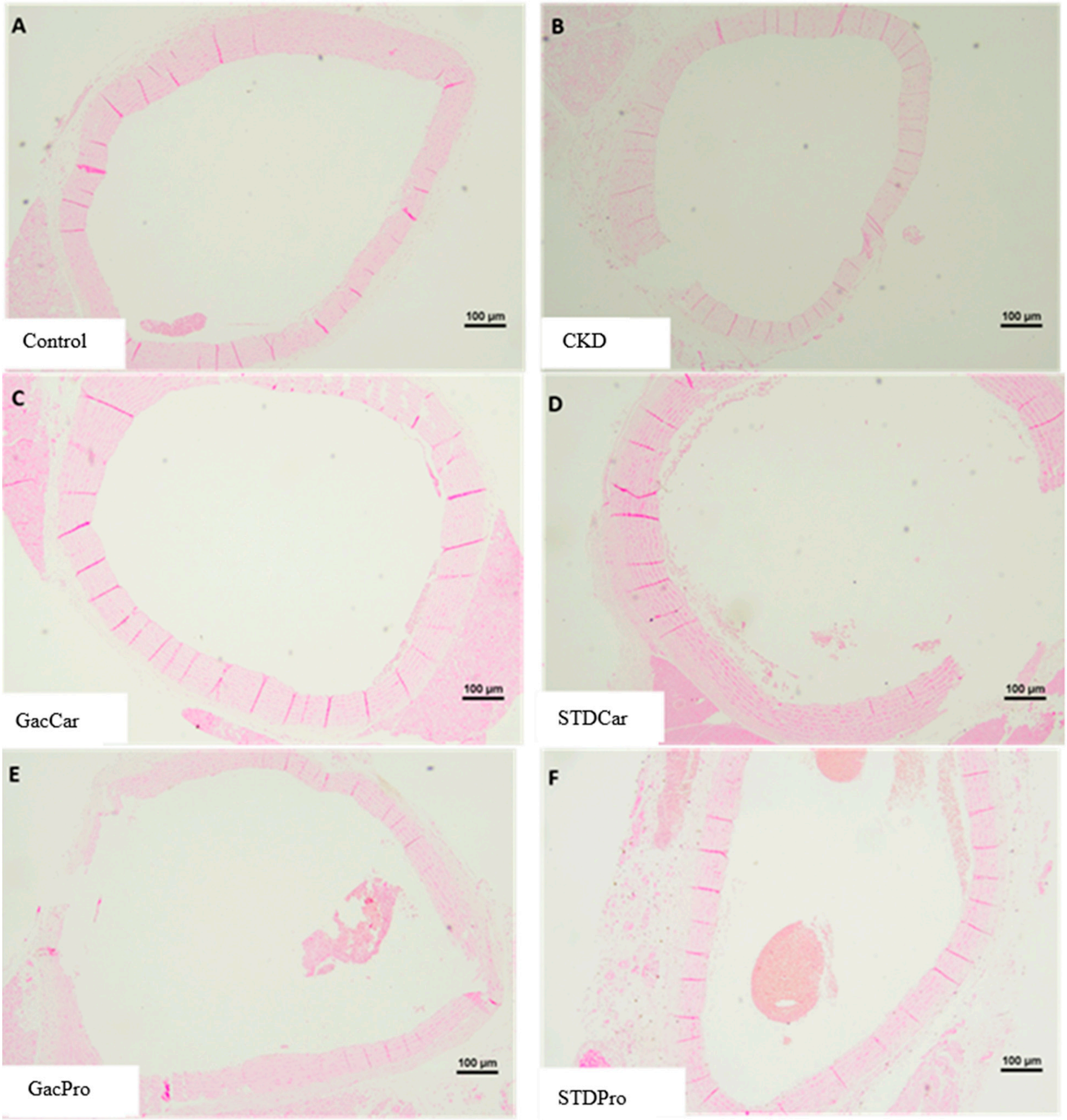

Figure S2: Pathology of aortic arch (von Kossa staining, 40X light microscopy)

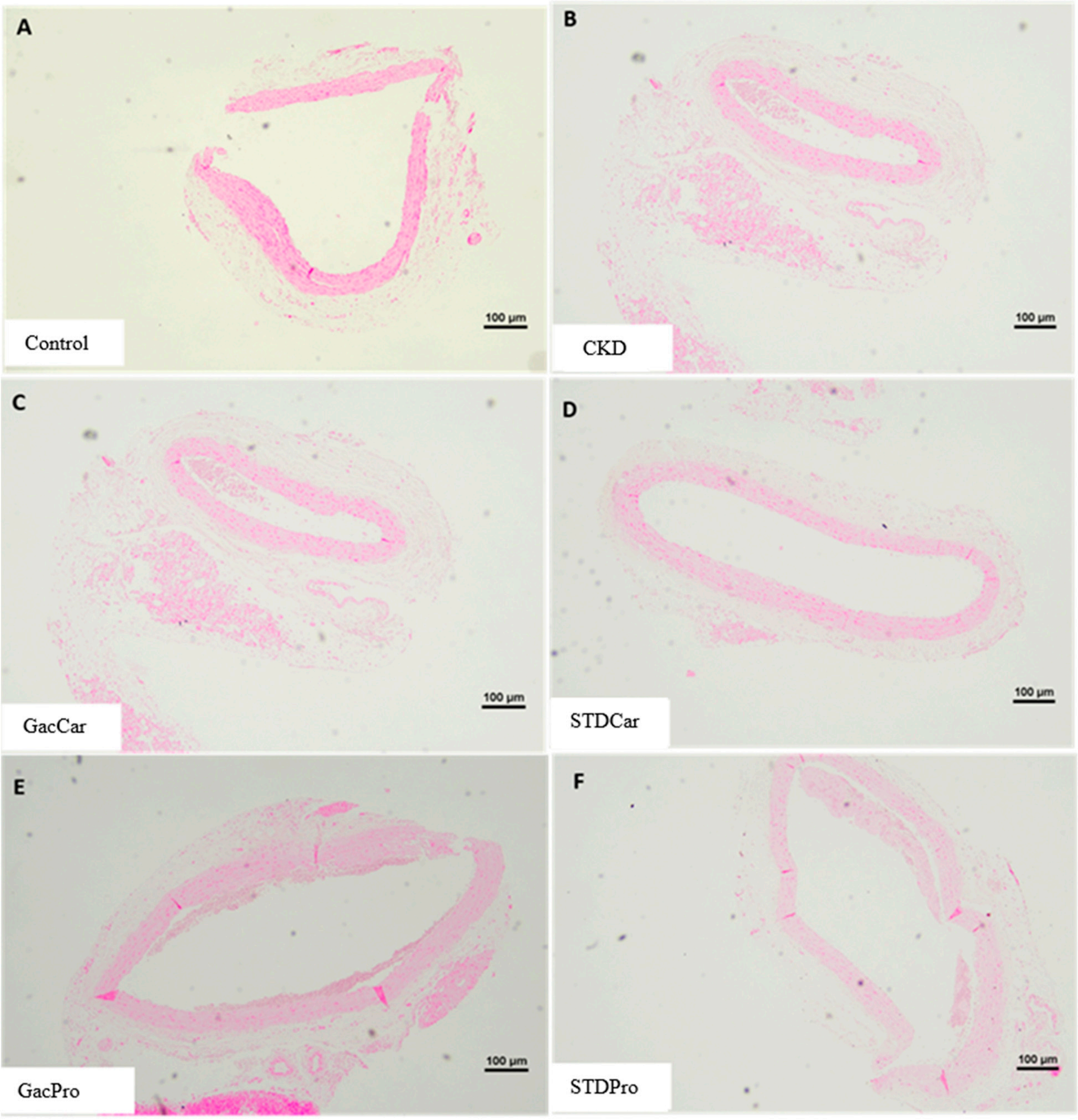

Figure S3: Pathology of abdominal aorta (von Kossa staining, 40X light microscopy)

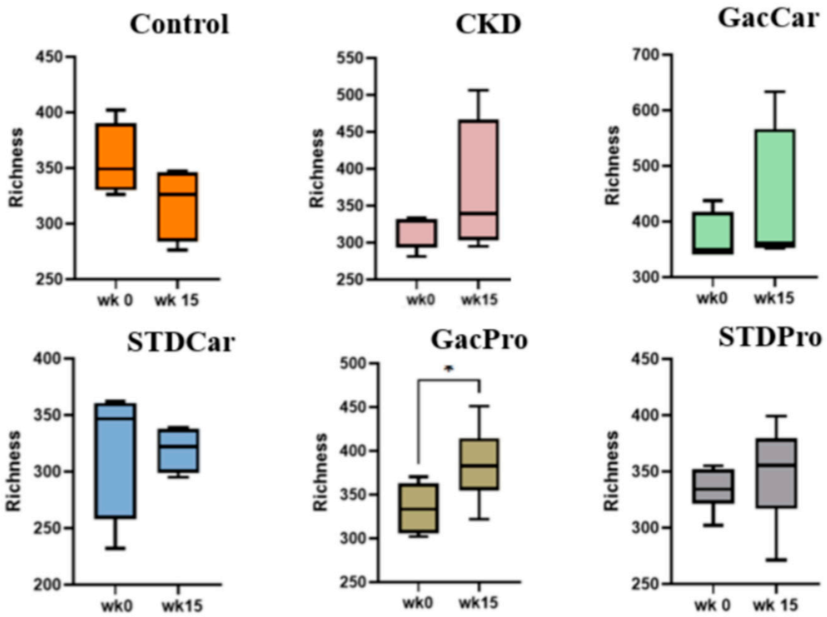

Figure S4: The comparison of fecal gut microbiota richness between week 0 vs week 15
